# Effect of Aromatic Amines on the Properties of Formaldehyde-Based Xerogels

**DOI:** 10.3390/gels6010008

**Published:** 2020-03-02

**Authors:** David Martin, Martin Prostredný, Ashleigh J. Fletcher

**Affiliations:** Department of Chemical and Process Engineering, University of Strathclyde, Glasgow, G1 1XJ, UK; david.martin.2015@uni.strath.ac.uk (D.M.); martin.prostredny@strath.ac.uk (M.P.)

**Keywords:** xerogel, Brunauer–Emmet–Teller (BET), Barret–Joyner–Halenda (BJH), resorcinol, formaldehyde, ammeline, melamine, melem

## Abstract

This study investigates the synthesis of formaldehyde-based xerogels using alternative aromatic precursors, with comparison to traditional resorcinol-formaldehyde analogues, in order to alter the chemical composition of the resulting gels. By replacing resorcinol with aromatic amine molecules, i.e., ammeline, melamine and melem, each expected to undergo similar reactions with formaldehyde as the substituted species, we found that for all substituted gels, at low additive contents, the gel structure was compromised and non-porous materials were formed, as opposed to the most abundant monomers, and therefore, these additives seem to act as impurities at low levels. Working towards higher additive contents, melem monomers exhibited low solubility (~5%), even at elevated temperatures, thereby limiting the range to which melem could act as a substitute, while melamine could be incorporated up to ~40% under acidic conditions, with enhanced microporosity over this range. Pure gels were successfully synthesised from ammeline, but their performance was inferior to resorcinol-formaldehyde gels, while melamine-formaldehyde analogues required acidic reaction conditions but shrank considerably on sub-critical drying, adversely affecting the gel properties and demonstrating their lack of potential as sorbents. This demonstrates the potential for the inclusion of aminated aromatics within resorcinol-based gel systems, however, only as partial substitutes and not complete replacements.

## 1. Introduction

Since the first porous organic materials were synthesised by Pekala in 1989 [1], using resorcinol and formaldehyde as precursors, other reagents have been utilised, in order to change the chemical character of the final products [2,3,4,5,6]. One family of such materials are aromatic amines, including melamine, ammeline, and melem, which can be used to introduce basic amino groups on to the material surface, leading to enhanced interactions with acidic gases, such as CO_2_ [2,3]. Such changes in surface functionalities may be useful for gas separation and storage, as well as offering new routes to further chemical modification of the surface, e.g., attachment of biologically relevant molecules [7,8,9].

It has been reported that, for high surface area materials, the micropore volume is predominantly beneficial for gas adsorption applications [10]. Therefore, it is advantageous to maximise the microporous surface area of such sorbent materials, for example in the well-established structures of resorcinol-formaldehyde (RF) and melamine-formaldehyde (MF) gels. Porous RF gels were first reported by Pekala in 1989 [1], synthesised using a base catalysed polycondensation reaction between R and F. Pekala adapted this process in 1992 [4] to form MF gels through a similar polycondensation reaction between M and F. In both cases, the base promotes the addition reaction between R/M and R to form substituted monomers that undergo self-condensation to create oligomeric chains that form clusters, which cross-link to form the final gel structure.

It has been well documented in the literature that varying reaction conditions, such as R to F molar ratio (R/F), R to catalyst (C) molar ratio (R/C), solids content and initial pH, can affect the physical properties of the final gel structure. Previous research has shown that these factors can be controlled to tailor the final gel structure, in terms of textural properties, to the specific needs of an end application. Sub-critical drying under a vacuum, following a solvent exchange step, is commonly used to produce xerogels [10], as it offers reduced cost compared to other drying techniques.

**Figure 1:**
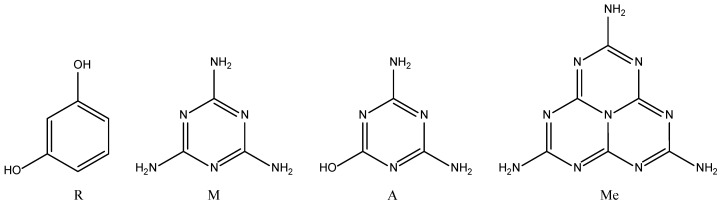
Reagents used in this study. From left to right: resorcinol (R), melamine (M), ammeline (A), and melem (Me).

This paper reports a number of synthetic options for the production of nitrogen enriched xerogels, including resorcinol-melamine-formaldehyde (RMF), melamine-formaldehyde (MF), ammeline-formaldehyde (AF), and melamine-melem-formaldehyde (MMeF) gels. The species selected to substitute resorcinol were each chosen as they were expected to offer similar chemical interactions with F (Figure 1), and to take part in polycondensation and cross-linking steps. The final materials were characterised in terms of textural properties to determine their potential in gas adsorption applications. 

## 2. Results and Discussion

### 2.1. Resorcinol–Melamine–Formaldehyde Gels

As discussed above, both RF [5,6,11] and MF [4,12] gels have been synthesised previously, leading to the production of a number of combined composites, i.e., RMF [2]. Procedurally, the RMF gels require pre-heating of the M-F-C solution, in order to fully dissolve the melamine into solution and allow its successful incorporation into the gel structure. Without this step, melamine forms a precipitate, which sediments at the bottom of the gel mixture.

**Figure 2:**
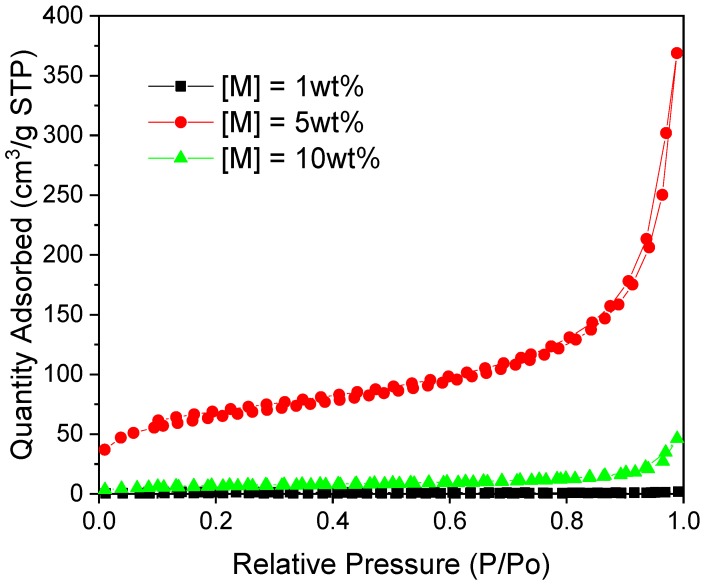
Nitrogen sorption isotherms for RMF gels prepared under basic conditions and varying melamine content.

In order to understand the effect of M substitution on RF gel formation, the R/C ratio was maintained at 100, and the percentage substitution of M was set at 1, 5 or 10%. Base catalysed RMF gels, created using Na_2_CO_3_, were synthesised in the presence of NaOH as an additional component, acting as a pH regulator, to further promote the reaction between M and F. While the resulting xerogels were opaque, as expected for their RF analogues, they demonstrated low surface areas compared with R/C 100 RF gels [11]. This implies that large clusters have formed during synthesis, which is supported by the wider average pore diameters. The nitrogen sorption isotherms for 5 and 10% M (Figure 2) exhibit a Type II shape, suggesting a macroporous or nonporous surface [13]. Higher gas uptakes for the 5% M sample suggests it is comprised of wide macropores, rather than being a nonporous material. There was little clear trend in total surface area with melamine content ([M]); however, the 5% sample did show appreciable textural properties compared to the other two samples (Table 1). The reason that the 5% sample stands out in comparison to 1% and 10%, could be caused by the interplay of initial structure formed during the gelation and curing step and subsequent structure collapse during subcritical drying. Further investigation of base catalysed RMF gels was not undertaken, as all samples showed comparatively low values for textural properties compared with standard RF gels, as well as RMF samples prepared under acidic conditions. Additionally, RMF materials prepared under basic conditions with M content between 1% and 10% could be further explored, to elucidate an optimal value of M content. 

**Figure 3:**
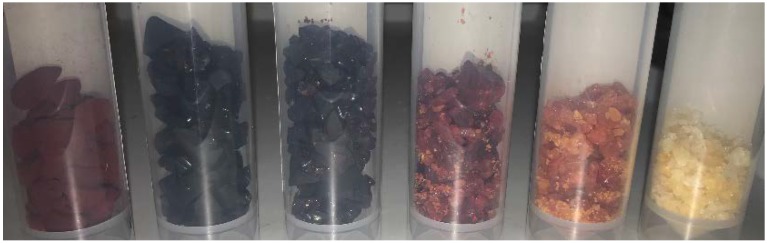
RMF gels prepared under acidic conditions with varying melamine content: from left to right [M] = 0%, 20%, 40%. 70%, 90%, and 100%.

As described in the methods section, there was enhanced solubilisation of the additive species under acidic conditions, allowing M to be incorporated into the final materials to a higher degree. At low M content, the textural character was similar to that observed for the base catalysed gel, with essentially non-porous character; such behaviour was also observed for M contents of 50% and higher, leaving a small operating window for materials development. As the M content increased, the gels became more transparent (Figure 3), suggesting that the cluster size decreases with increasing M content. The images presented in Figure 3 show a clear transition between pure RF gels and pure MF gels was observed. The lower M content gels do not appear to have been affected as severely by shrinkage under vacuum drying as the higher M content counterparts, estimated as ~40% volume reduction for the lower M contents; as discussed below, this contrasts with the pure MF gels, which shrank considerably and exhibited low textural properties as a consequence of pore collapse. This suggests that R acts as a ‘backbone’ structure to the M, allowing the structure of the gel, and resulting pores, to withstand the drying process. RMF gels offer potential as porous solids for carbon capture applications, as a result of their high surface areas combined with the high degrees of inherent nitrogen functionality.

**Figure 4:**
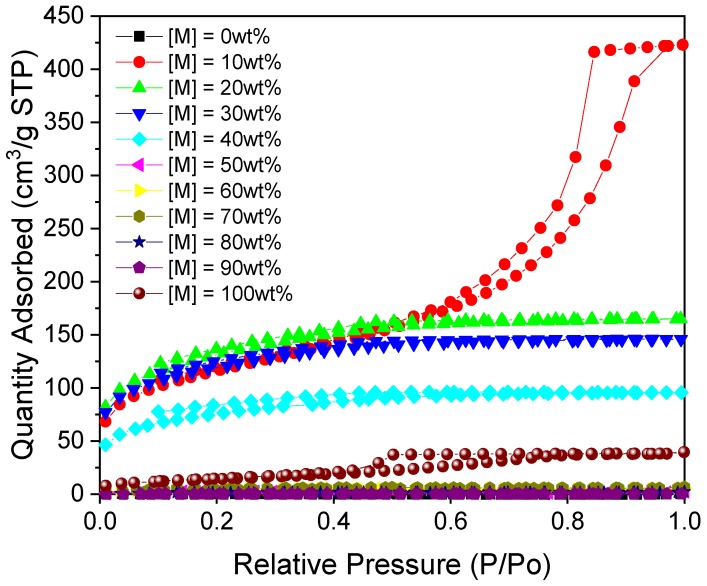
Nitrogen sorption isotherms for RMF gels prepared under acidic conditions (pH 2) with varying melamine content (R/C = 100).

From the isotherms presented in Figure 4, the RMF gel with 10% M exhibited the highest values of nitrogen uptake and a Type IV(a) isotherm shape with a Type H2(b) hysteresis loop, suggesting mesoporous character with a wider distribution of pore necks, accompanied by network effects (e.g., pore-blocking or percolation) [13]. On the other hand, the isotherms obtained for samples with [M] = 20% to 40% are similar to a Type I(b) isotherms, common for microporous materials with relatively small external surface areas. The less steep shoulder, present at lower values of P/P_0_ for each of these isotherms, suggests the presence of micropores with a wide distribution of sizes [13].

In addition to total surface area, the performance of the RMF gels was modified by inclusion of M into the system (Table 2). For M contents of 20%–30%, the gels exhibited comparatively enhanced micropore volumes (0.089 and 0.086 cm^3^/g, respectively), which represented a high degree of the total pore volume available (0.255 and 0.226 cm^3^/g). This offers an improvement on previously reported results from our group [2] and, within a carbon capture context, an M content of ~30%, offers an optimal combination of total surface area, microporosity and nitrogen functionality for this family of materials. Interestingly, it seems that the optimal value of M content is different for RMF gels prepared under acidic conditions, compared to the samples discussed above prepared with a basic catalyst.

### 2.2. Melamine-Formaldehyde Gels

The gelled structures of MF systems were transparent with a light blue tinge, which is likely a result of Raleigh scattering caused by the MF clusters formed within the gel structure [4]. As mentioned above, the MF materials did not successfully gel using the base catalysed reactions, and a precipitate was observed to have formed at the bottom of the jar. Thus, acidic conditions were required for gel formation in these systems. The type of polycondensation bridges formed has been shown to be pH dependent: at low basic pH ranges (7–8) methylene bridges are favoured, while at higher pH (≥9), ether bridges are more dominant [14]. It is important to note that Johromi’s work focussed on the production of MF resins, not xerogels [14]. In this study, the initial pH of the reaction solution was between 8 and 11, with higher values obtained for lower R/C ratios. As a consequence of the formation of resins at basic pH, the initial pH of the MF systems was adjusted to more acidic conditions (pH 2) to increase M dissolution, via substituted derivatives.

The total surface area increased with an increasing M/C ratio up to 100, before decreasing at higher values. All samples showed small levels of porosity and negligible microporosity within this, which is also reflected in the low surface areas obtained for these samples. These figures are significantly lower than values stated in literature for MF aerogels [12], reinforcing the fact that vacuum drying can be detrimental to gel porous structure. The isotherms obtained are generally Type IV in shape, with a H2a hysteresis loop. Consequently, the pore dimensions remain consistent, at 3–4 nm, across the suite of samples, at pH 2.

All MF xerogels exhibited considerable shrinkage after vacuum drying to form hard, resin-like structures. This implies that the pore structure of these materials is affected to a greater degree by the drying process than RF gels.

**Figure 5:**
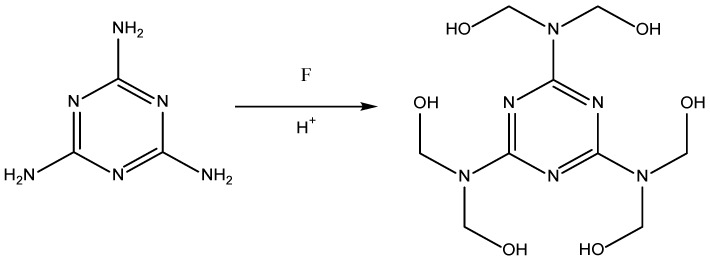
Anticipated reaction of melamine and formaldehyde under acidic conditions, forming hydroxymethyl derivatives.

**Figure 6:**
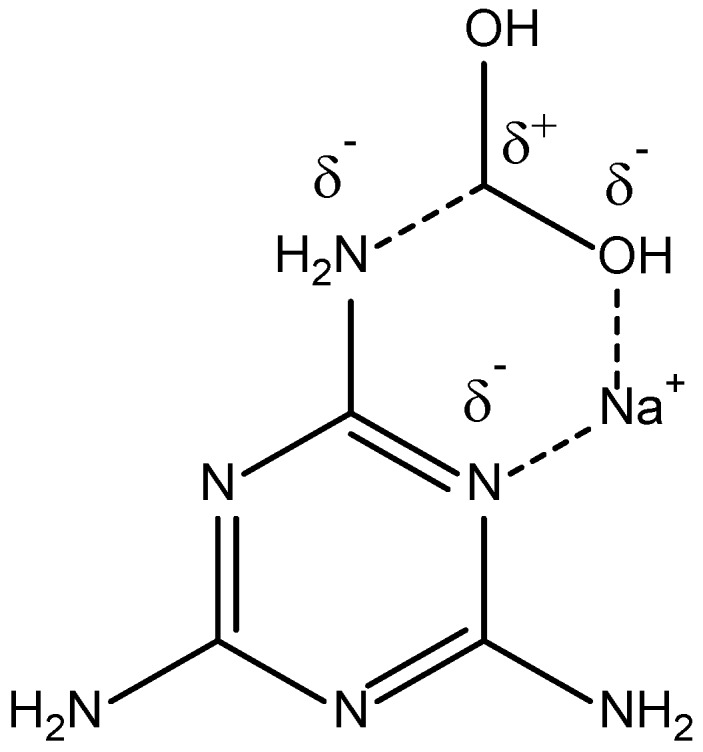
Proposed intermediate species formed during reaction of melamine with formaldehyde.

**Figure 7:**
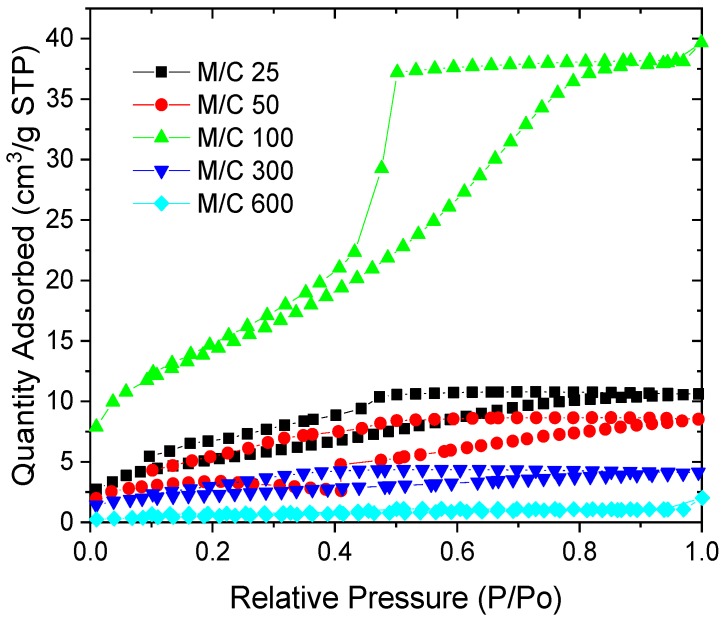
Nitrogen sorption isotherms for MF gels prepared with varying M/C ratio, under acidic conditions (pH 2).

Under acidic conditions, the amine groups of M can undergo condensation reactions with F, which is present within the solution. Each amine group can react with up to two F molecules, leading to up to six reaction sites on an individual M molecule (Figure 5). In theory, the MF reaction, as also expected for RF gels, may undergo a similar stabilisation step (Figure 6), as previously proposed for phenolic systems [15]. The intermediate structure results from the differences in partial charges on the nitrogen atoms of melamine, potentially further stabilised by the presence of the metal cations, contributed by the catalyst. This stabilisation by sodium ions could be one of the factors influencing the difference in textural characteristics of materials prepared with different amounts of sodium carbonate (Figure 7 and Table 3). In addition, a common factor influencing all xerogel materials is the interplay between structure growth during the initial synthesis process and resulting mechanical resistance to drying conditions. 

### 2.3. Ammeline-Formaldehyde Gels

The effect of the catalyst ratio on AF gels was investigated by synthesising gels with A/C ratios of 100, 300 and 500. It should be noted that for A/C 500, the low volume of the gel mixture (30 cm^3^), in tandem with the small amount of catalyst required, resulted in a higher degree of uncertainty in weighing of the catalyst. For all AF gels syntheses, heating the reaction mixture initially produced a milky white solution, which persisted over all stages of the synthesis. The resulting final gels, for all A/C ratios studied, were similar in appearance, being soft materials, easily cut with a blunt edge, which resembled the forms typically observed for high R/C ratios of RF gels. The textural characteristics obtained for the suite of AF xerogels was markedly different to those of RF gels with similar catalyst loadings. The surface areas were significantly reduced, ~30 m^2^/g, but were consistent over all A/C ratios used, while the micropore volumes were negligible (Table 4).

**Figure 8:**
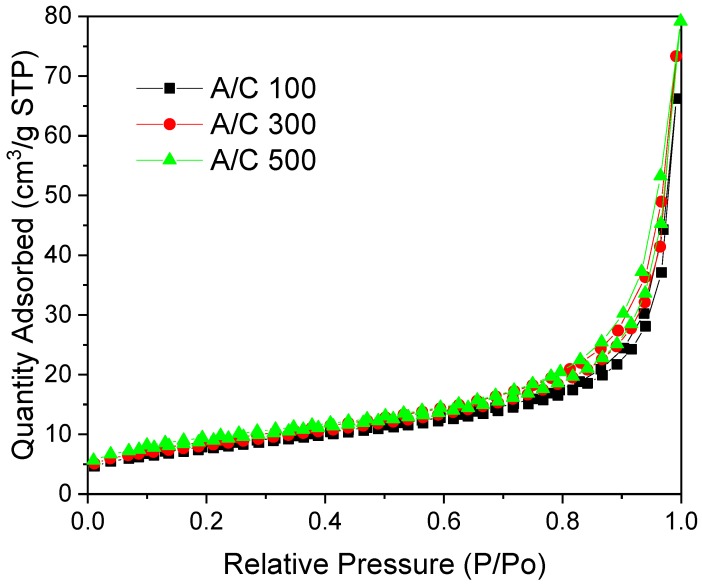
Nitrogen sorption isotherms for AF xerogels with varying A/C ratio, synthesised under basic conditions.

In contrast with RF gels obtained over such a wide catalyst range, the pore width was relatively constant for each AF gel (Table 4). The isotherms obtained showed no plateau at higher relative pressures (Type II [13]), indicating that the materials are non-porous or exhibit predominantly macroporous structures (Figure 8). The difference in chemical structure of A and M could be the reason for the different character of the final materials. While M contains three amine groups, A contains only two, with one replaced by a hydroxy group. Since the hydroxy group does not take part in the condensation reactions with F, this reduces the potential cross-linking ability of this reagent. Consequently, based on the criteria outlined above, AF xerogels, as prepared here, offer little scope for gas adsorption applications. However, it is worth mentioning that the materials studied in this work are dried subcritically, leading to a significant structure collapse. Therefore, these materials could offer more favourable textural character in the form of costlier, and thus less industrially viable, aerogels.

### 2.4. Melem Composite Gels

As mentioned in the introduction, the inclusion of nitrogen-rich aromatic species in place of R in the synthesis of RF gels can lead to favourable adsorption interactions between the nitrogen moieties and the acidic CO_2_ gas. Melem (Me) is a molecule that offers significant nitrogen content, having seven ring nitrogens and three amino functionalities, and may be considered as an expanded homologue of melamine, offering a higher molar ratio of nitrogen per mole of sorbent. The central nitrogen of the tri-s-triazine ring contributes to the conjugated stabilisation of the molecule and results in a planar conformation overall. Given the larger, bulkier structure of Me, compared with M, the molecule may also offer benefit in the vacuum during stages, where MF gels have exhibited significant shrinkage due to collapse of their pore structure, due to the relative rigidity of the planar conformation.

**Figure 9:**
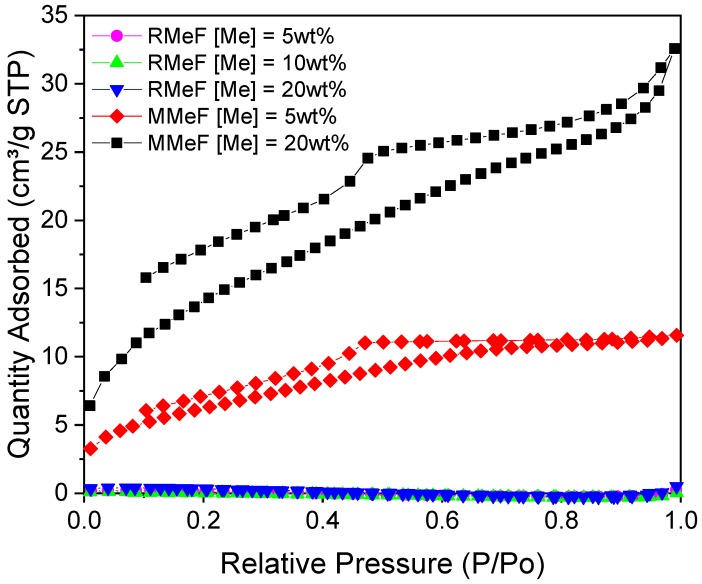
Nitrogen sorption isotherms for melem composite gels with R/C and M/C ratio 100.

Syntheses using Me were challenging, due to the low reactivity and solubility of Me in water, and this presented further issues to those already encountered with M, and required additional modifications to the experimental procedure. As a result of the planar structure of the ring formation, the nucleophilicity of the amino groups is low [16], and reactions involving the amino groups as nucleophilic reagents are very rare [17]. This previous work informed the modifications made here, where the reaction between Me and F was promoted at elevated temperatures (80–130 °C), and ~90 °C was used in this study as the reaction medium is water. Addition to MF gel systems was undertaken as the chemistry of the aromatic molecules is similar and the reaction protocol would be common for the two species. Preliminary experiments on RMeF gels produced non-porous structures for both basic and acidic conditions (<5 m^2^/g), which, given the scope of materials development undertaken here, were considered of little value (Figure 9 and Table 5).

Low Me content in MMeF gels significantly impacted the textural properties observed for these materials (Figure 9 and Table 5). Note that the failure of the isotherms to close may be related to the sample size used for these measurements, however, we were limited by the maximum mass for the equipment, due to a combination of low density and low surface area. Increasing the Me content was limited, due to its poor reactivity and solubility within the reaction system, with a precipitate formed at the bottom of the reaction vessel, which suggests that Me has not fully reacted and has limited presence in the final gel structure. MMeF gels appeared similar in appearance to standard MF gels and the Me did not seem to have such a detrimental effect on the gel structure for MMeF gels in comparison to RMeF gels. Viable gels were created using 5% and 20% Me, the former gave a reduced surface area of 20 m^2^·g^−1^, while the 20% sample showed similar character to the M/C 100 MF gel, with a surface area of ~50 m^2^/g. This indicates that Me can be successfully incorporated into the MF framework, creating composite materials with comparable textural properties, but the additional benefit of nitrogen functionalised surfaces. Similar to AF materials, superior materials could be potentially obtained using supercritical drying, however, this was not within the scope of the work reported here.

## 3. Conclusions

The work undertaken here shows that the low solubility and reactivity of melamine and melem made it difficult to incorporate them into gel structures. Issues with melamine were successfully resolved by shifting the pH of the reaction into the acidic range, promoting the formation of more soluble derivatives. Such methods were less successful for melem, due to its higher molecular mass and lower reactivity of amino functionalities as a result of ring stabilisation effects, limiting its incorporation to ~20%. Interestingly, all composite gel systems showed significant impact on the textural characteristics of the gels with the incorporation of low quantities of a third species. This may be due to disruption of the chemical interactions of the dominant aromatic with formaldehyde, without the additional benefit of sufficient amounts of the third species to competitively participate in the reaction itself. Ammeline-formaldehyde gels were synthesised successfully, but they demonstrated reduced porous character compared with standard resorcinol-formaldehyde gels. Similarly, melamine-formaldehyde gels were poorer in textural development, but this was a result of significant shrinkage during vacuum drying, due to pore collapse. As expected, and in line with results observed in previous studies, there is a high degree of correlation between specific surface area and micropore volume, which is central to gas adsorption applications, and this positive relationship arises from the interconnectivity of these two textural properties. This study focusses on gel synthesis, and while these textural parameters indicate available adsorption capacity for suitable adsorptives, it is not possible to deduce their potential for a specific gas adsorption application without subsequent adsorption analysis, as this involves an interplay between surface moieties, adsorption capacity and suitability of the pore sizes available for the target adsorptive. Introducing nitrogen-rich precursors into these composite gel systems would increase the nitrogen functionalisation of the resulting materials, and the gels developed here offer insight into future approaches to obtain such sorbents, which may require additional modification and optimisation of the synthesis route.

## 4. Materials and Methods 

### 4.1. Gel Formation

All gel samples were prepared using a procedure analogous to that for RF gels, previously reported in the literature [11], with slight alterations due to reagent solubility, as discussed below. Resorcinol (R, Sigma Aldrich, Gillingham, UK, Reagent Plus, 99%), melamine (M, Sigma Aldrich, Gillingham, UK, 99%), ammeline (A, TCI, Oxford, UK, ≥95%), and melem (Me) were used as aromatic reagents in this work. All chemicals were used as received from the supplying company as detailed for each component, apart from melem, which was synthesised according to the procedure described below, and deionised water was prepared in-house (Millpore Elix^®^ 5 with Progard^®^ 2, Merck, Watford, UK). In order to adjust the reaction mixture pH, sodium carbonate (Sigma Aldrich, anhydrous, ≥99.5%) and sodium hydroxide (Fisher Scientific, Loughborough, UK, Analytical reagent grade, 98.8%) were used for base-catalysed gelation, while hydrochloric acid (Sigma Aldrich, 37%) was used for reactions under acidic conditions. 

All reaction solutions were prepared using 20w/v% solids content, taking into account all aromatic reagents (R, M, A, Me), catalyst, and F. The total liquid volume used, unless otherwise stated, was 60 cm^3^, made up of water and methanol, which is contributed by the formalin solution used as a source of F. The ratios of catalyst to R were reported on a molar basis (R/C), with values in the range 100–600, unless otherwise stated. The ratio of R and F (R/F) was set to 0.5, unless otherwise stated. Generally, all the solids were initially dissolved in a glass jar with a magnetic stirrer in a premeasured volume of deionised water, followed by the addition of the required volume of formalin solution (SigmaAldrich, 37% F in water, containing 10%–15% methanol as a polymerization inhibitor). Due to the low solubility of M and Me in the reaction solution, R was added after dissolution of M or Me, with increased solubility of their respective hydroxymethyl derivatives.

The solids content remained fixed for synthesis of RMF gels while changing the ratio between R and M. Due to the low solubility of M in the reaction mixture, and increased number of reaction sites with F, the R/F ratio for these materials was set to 0.33. Furthermore, in order to promote dissolution of M, prior to the addition of F, the mixture was heated to 70 °C, after the addition of M and catalyst, and stirred for 15 min until a clear solution was formed. Afterwards, the mixture was allowed to cool to ambient temperature, followed by the addition of R and subsequent stirring for a further 30 min. 

AF synthesis followed a similar procedure to that for RF gels [11], due to the sufficient solubility of A in the reaction mixture, while MF gels could not be successfully synthesised using base catalysis, with a lack of gelation and phase separation to give a white precipitate at the bottom of the reaction vessel. Consequently, acidic conditions were required to create MF gels, with the reaction solution titrated with a 2 M solution of HCl to achieve a pH of 2 (measured using an InLab^®^ Expert Pro electronic pH meter from Mettler Toledo, Leicester, UK), which also allowed a higher degree of M to be incorporated into the reaction mixture. Similar to the RMF gels, due to the poor solubility of both M and Me, the [M+Me]/F ratio was set to 0.3 and the solution was heated to 90 °C and the addition of F, in order to dissolve M and Me by creating the more soluble hydroxymethyl derivatives.

After all solids were dissolved, stirrer bars were removed from the reaction mixtures and the jar lids were hand-tightened, before transference to an oven (Memmert UFE400, Schwabach, Germany), pre-heated to 85 °C. In order to allow sufficient time for gelation and curing of the samples, as per the procedure used for RF gels [12], the samples were left in the oven for 3 days. After the curing period, the jars were taken out of the oven and allowed to cool to ambient temperature.

Prior to the drying stage, a solvent exchange step was performed using acetone (SigmaAldrich, ≥ 99.5%), in order to replace water contained within the gel pore structures; reducing potential structure collapse and material shrinkage, during drying that would result from high surface tension of water. The gel samples were first cut into smaller pieces (~1 cm) and washed with acetone to remove excess water from the gel and jar surfaces. After the initial amount of acetone was drained, 80 cm^3^ of fresh acetone was added to the sample, the jar lid was replaced and wrapped in paraffin film to minimise acetone losses during the exchange period. The sealed jars were put on a shaker unit (VWR 3500 Analog Orbital Shaker, Lutterworth, UK), to promote acetone mixing, for 3 days. If a sample was deemed too soft, as determined during the cutting step, the jar was left on a bench top, and periodically mixed gently, preventing mechanical damage to the gel structure. The acetone was drained and replaced with 80 cm^3^ of fresh solvent on each successive day of the exchange period.

After 3 days of solvent exchange, the acetone was drained and the samples were moved to a vacuum oven (Towson and Mercer 1425 Digital Vacuum Oven, Stretford, UK). The oven was set to 85 °C in order to match the curing temperature, and mitigate any structural changes due to thermal effects. The samples were dried under vacuum for 2 days, after which the xerogels were transferred to labelled sample tubes for storage.

### 4.2. Melem Synthesis

Melem was produced via the method outlined by Sattler et al. [18] Ceramic crucibles were loaded with M powder (Sigma Aldrich, 99%) and wrapped in aluminium foil to contain the powder inside the crucibles, before placing the loaded crucibles in a furnace (CARBOLITE 15/75/450). In order to prevent oxidation, the reaction was performed in an inert atmosphere with nitrogen gas (BOC, oxygen-free nitrogen gas) flow set at 60 cm^3^/min. First, the furnace was heated to 450 °C at a heating rate of 10 °C/min, followed by an isothermal stage at 450 °C for 5 h, to allow enough time for the reaction to occur. After 5 h, the heating was switched off and the system was allowed to cool to room temperature overnight, maintaining the flow of nitrogen gas inside the furnace. The resulting product mixture was placed in a round bottom flask and 100 cm^3^ of deionised water was added for every 1 g of product powder. The mixture was boiled under reflux for 3 h in order to remove water-soluble impurities, as suggested in [18]. Purified product, in the form of a beige powder, was extracted from the mixture by vacuum filtration and left to dry at ambient temperature. The colour change from white M powder to beige melem product is consistent with the description by Jurgens et al. [19] In order to confirm the presence of melem in the product, FTIR spectra of the product were obtained (see Appendix A), and notably peaks at 802, 1469 and 1606 cm^–1^ were confirmed [19].

### 4.3. Sample Characterisation

The prepared gels were analysed using nitrogen sorption measurements in order to determine their textural properties. Nitrogen sorption was carried out at −196 °C using a Micromeritics ASAP 2420 surface area and porosity analyser. In order to remove any previously adsorbed species on the sample surface, a degas process was followed, involving outgassing under vacuum below 10 µmHg at 50 °C for 30 min and then 110 °C for 2 h. For analysis, a 40 pressure point adsorption and 30 pressure point desorption cycle was used. In the cases where the total surface area of the analysed sample was less than 100 m^2^, a volume displacement insert was used to reduce measurement errors, as per the guidelines of the instrument manufacturer. All samples were analysed for surface area [m^2^/g] using the Brunauer–Emmett–Teller (BET) [20] theory, applying the Rouquerol correction [21] for microporous samples; total pore volume [cm^3^/g]; micropore volume [cm^3^/g] using the t-plot method [22]; and average pore size [nm] from the Barrett–Joyner–Halenda method [23].

## Figures and Tables

**Table 1:** Textural properties of RMF gels prepared under basic conditions and different melamine content.

[M] %	S_BET_ [m^2^/g]	V_T_ [cm^3^/g]	V_µ_ [cm^3^/g]	$\bar{\varphi }$ [nm]
1	3	-	-	37
5	200	0.57	0.034	15
10	20	0.07	0.003	19

S_BET_—surface area from Brunauer–Emmet–Teller (BET) analysis; V_T_—total pore volume determined from adsorption at p/p_o_ ~1; Vµ—micropore volume determined using *t*-plot method; $\bar{\varphi }$—average pore width from Barrett–Joyner–Halenda (BJH) analysis. Errors are omitted from the table as all values are reported to an accuracy less than the largest error for each variable.

**Table 2:** Textural properties of RMF gels prepared under acidic conditions (pH 2) with varying melamine content (R/C = 100).

[M] %	S_BET_ [m^2^/g]	V_T_ [cm^3^/g]	V_µ_ [cm^3^/g]	$\bar{\varphi }$ [nm]
1	1	-	-	-
10	400	0.65	0.058	8
20	500	0.26	0.089	3
30	400	0.23	0.086	3
40	300	0.15	0.052	3
50	5	-	0.002	2
60	10	0.01	0.004	10
70	20	0.01	0.003	4
80	4	-	-	6
90	-	-	-	-

S_BET_—surface area from BET analysis; V_T_—total pore volume determined from adsorption at p/p_o_ ~1; V_µ_—micropore volume determined using *t*-plot method; $\bar{\varphi }$—average pore width from BJH analysis. Errors are omitted from the table as all values are reported to an accuracy less than the largest error for each variable.

**Table 3:** Textural properties of MF gels with varying M/C ratio, under acidic conditions (pH 2).

M/C Ratio	S_BET_ [m^2^/g]	V_T_ [cm^3^/g]	V_µ_ [cm^3^/g]	$\bar{\varphi }$ [nm]
25	20	0.016	0.001	3
50	10	0.013	0.001	3
100	50	0.061	-	4
300	7	0.006	-	3
600	1	0.003	-	4

S_BET_—surface area from BET analysis; V_T_—total pore volume determined from adsorption at p/p_o_ ~1; Vµ—micropore volume determined using *t*-plot method; $\bar{\varphi }$—average pore width from BJH analysis. Errors are omitted from the table as all values are reported to an accuracy less than the largest error for each variable.

**Table 4:** Textural properties of AF xerogels with varying A/C ratio, synthesised under basic conditions.

A/C Ratio	S_BET_ [m^2^/g]	V_T_ [cm^3^/g]	V_µ_ [cm^3^/g]	$\bar{\varphi }$ [nm]
100	30	0.122	0.003	19
300	30	0.102	-	17
500	30	0.113	-	17

S_BET_—surface area from BET analysis; V_T_—total pore volume determined from adsorption at p/p_o_ ~1; V_µ_—micropore volume determined using *t*-plot method; $\bar{\varphi }$—average pore width from BJH analysis. Errors are omitted from the table as all values are reported to an accuracy less than the largest error for each variable.

**Table 5:** Textural properties of melem composite gels with R/C and M/C ratio 100.

Sample Name	S_BET_ [m^2^/g]	V_T_ [cm^3^/g]	V_µ_ [cm^3^/g]	$\bar{\varphi }$ [nm]
RMeF [Me] = 5%	-	-	-	-
RMeF [Me] = 10%	-	-	-	-
RMeF [Me] = 20%	-	-	-	-
MMeF [Me] = 5%	20	0.018	-	4
MMeF [Me] = 20%	50	0.050	-	5

S_BET_—surface area from BET analysis; V_T_—total pore volume determined from adsorption at p/p_o_ ~1; V_µ_—micropore volume determined using *t*-plot method; $\bar{\varphi }$—average pore width from BJH analysis. Errors are omitted from the table as all values are reported to an accuracy less than the largest error for each variable.

**Figure A1:**
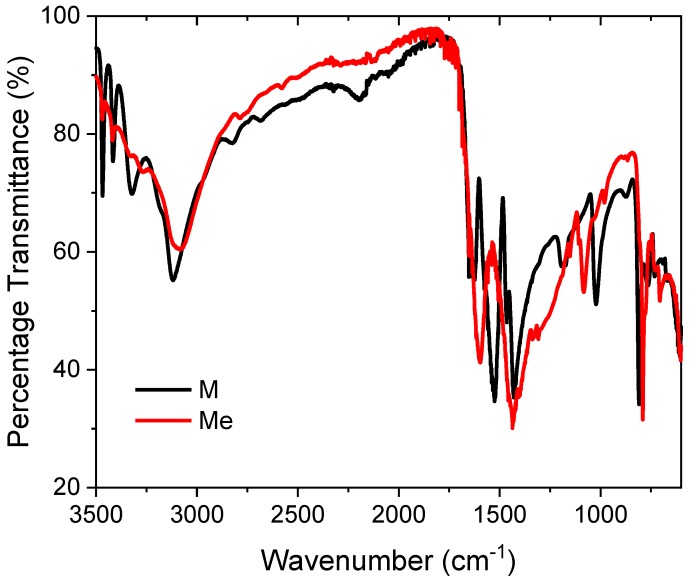
FTIR spectra of melamine (M) and melem (Me).

## Appendix A

The feedstock (M0 and resulting product (Me) were analysed by Fourier transform infrared spectroscopy (FTIR) for surface characterization (ABB Instrument MB3000 series S FTIR spectrometer with internal reflection elemental of diamond; each spectrum was the average of 86% scans with spectra resolution 4 cm^−1^) to confirm the transformation of M to Me in the reaction detailed above. The spectra obtained show notable differences for the two substances (Figure A1), especially in the range of wavenumbers 1000–2000 cm^−1^. Notably, peaks in the regions around wavenumbers 790, 1080, 1580, and 3000–3300 cm^−1^ offer confirmation of Me production in comparison with the work by Jurgens et al. [20]
